# A novel biosensor to monitor proline in pea root exudates and nodules under osmotic stress and recovery

**DOI:** 10.1007/s11104-020-04577-2

**Published:** 2020-06-04

**Authors:** María I. Rubia, Vinoy K. Ramachandran, Cesar Arrese-Igor, Estíbaliz Larrainzar, Philip S. Poole

**Affiliations:** 1 Institute for Multidisciplinary Research in Applied Biology-IMAB, Universidad Pública de Navarra, Campus Arrosadia, Pamplona, 31006 Spain; 2 Department of Plant Sciences, University of Oxford, Oxford, OX1 3RB UK

**Keywords:** Rhizosphere, Proline dehydrogenase, Water deficit, Salt stress, Rhizobium, Symbiosis

## Abstract

**Background and aims:**

Plant and bacteria are able to synthesise proline, which acts as a compound to counteract the negative effects of osmotic stresses. Most methodologies rely on the extraction of compounds using destructive methods. This work describes a new proline biosensor that allows the monitoring of proline levels in a non-invasive manner in root exudates and nodules of legume plants.

**Methods:**

The proline biosensor was constructed by cloning the promoter region of pRL120553, a gene with high levels of induction in the presence of proline, in front of the *lux* cassette in *Rhizobium leguminosarum* bv. *viciae*.

**Results:**

Free-living assays show that the proline biosensor is sensitive and specific for proline. Proline was detected in both root exudates and nodules of pea plants. The luminescence detected in bacteroids did not show variations during osmotic stress treatments, but significantly increased during recovery.

**Conclusions:**

This biosensor is a useful tool for the *in vivo* monitoring of proline levels in root exudates and bacteroids of symbiotic root nodules, and it contributes to our understanding of the metabolic exchange occurring in nodules under abiotic stress conditions.

**Electronic supplementary material:**

The online version of this article (10.1007/s11104-020-04577-2) contains supplementary material, which is available to authorized users.

## Introduction

Drought and salinity stress are some of the environmental factors most affecting plant growth and crop yield worldwide. In order to counteract the negative effects of osmotic stresses, plant and bacteria are able to synthesise osmoprotectant compounds to maintain cell viability. The amino acid proline, being highly soluble in water and a scavenger of reactive oxygen species, has been thought to provide protection under salt and water-deficit stresses (Aspinall and Paleg 1981; Hasegawa et al. 2000; Szabados and Savouré 2010; Verdoy et al. 2006). In plants, proline catabolism is mediated by two enzymes; proline dehydrogenase (ProDH) producing pyrroline-5-carboxylate (P5C) from proline, and delta-1-pyrroline-5-carboxylate dehydrogenase (P5CDH), which converts P5C to glutamate (Szabados and Savouré 2010). In bacteria, however, both steps are catalysed by a single polypeptide encoded by the gene *putA*, whose expression is regulated by *putR* and is induced in response to proline (Jimenez-Zurdo et al. 1997; Keuntje et al. 1995; Kohl et al. 1988). Besides its role as an osmoprotectant, proline catabolism has been also suggested to serve as an energy, carbon and nitrogen source under environmental stress conditions (Lee et al. 2009; Tanner 2008; van Overbeek and van Elsas 1995; Vives-Peris et al. 2018). Additionally, proline exudation has been shown to have a chemotactic effect in alfalfa roots (Bais et al. 2006; Webb et al. 2014). Monitoring proline utilisation in both plant and bacterial systems is a first key step towards understanding the multiple roles of this molecule under osmotic stress situations.

The rhizosphere is the nutrient-rich zone of soil in close proximity with the plant root system where microbial communities depend on the release of root exudates (Turner et al. 2013). Plant root exudates are composed of a great variety of primary and secondary metabolites, including low-molecular weight compounds such as sugars, amino acids and organic acids, as well as high-molecular weight molecules such as mucilage and proteins (Bais et al. 2006; Oburger and Jones 2018). The different molecules present in root exudates can mediate both positive and negative interactions in the rhizosphere (Huang et al. 2014; Olanrewaju et al. 2019). Focusing on the former, the symbiosis established between plants of the *Leguminosae* family and a group of alpha-proteobacteria named rhizobium has been widely studied. During this interaction, rhizobia are able to infect root cells through a complex signal exchange process, which requires the transcriptional reprogramming of roots cells to develop an organ specialized in nitrogen fixation named the nodule. The symbiosis between pea (*Pisum sativum*) plants and *Rhizobium leguminosarum* bv. *viciae* bacteria is a well-established model system to understand this plant-microbe interaction (Oldroyd et al. 2011; Udvardi and Poole 2013) and has been effectively used to analyse the effect of root exudates in bacterial gene expression (Ramachandran et al. 2011). This transcriptomic analysis led to the identification of a number of bacterial genes specifically induced in response to certain solutes. Cloning the promoter regions of such genes upstream of the *lux* operon, Pini et al. (2017) generated a suite of luminescence-based bacterial bioreporters for the specific detection of metabolites in the rhizosphere. These biosensors allow real time monitoring of the release of a number of compounds including sugars, polyols, organic acids and amino acids in a non-destructive semi-quantitative manner, avoiding possible artefacts associated with other methodologies (Oburger and Jones 2018; Rilling et al. 2019, and references therein). Plants are grown on plates and, upon inoculation of the specific biosensor, the presence and the abundance of a specific compound can be monitored over time using a photon counting CCD camera.

In the current work, we extend this elite set of biosensors by describing a new *lux* biosensor for the detection of the amino acid proline. The construct relies on the expression of the *lux* reporter driven by the promoter of the pRL120553, a gene located in the proximity to the gene *putA*, responsible for proline catabolism in gram-negative bacteria (Jiménez-Zurdo et al. 1995; Liu et al. 2017). We monitored the levels of luminescence of the biosensor in pea roots and during nodulation both under optimal growth conditions and upon the application of water-deficit or salt stress. Our results show that, in bacteroids, proline accumulation does not occur during the stress phase, but during recovery, once optimal plant growth conditions are re-established.

## Materials and methods

### Bacterial strains and growth conditions

The bacterial strains and plasmids used in this study are listed in Table 1. *R. leguminosarum* bv. *viciae* 3841 strains were grown at 28 °C in tryptone yeast agar or broth (Beringer 1974) or universal minimal agar supplemented with 30 mM pyruvate and 10 mM ammonium chloride as the carbon and nitrogen sources, respectively. Universal minimal salt medium (UMS) is based on the acid minimal salts (AMS; Poole et al. 1994) medium with the addition of EDTA-Na_2_ (1 µM), CoCl_2_O∙6H_2_O (4.2 µM), FeSO_4_O∙7H_2_O (0.04 mM), and CaCl_2_O∙2H_2_O (0.51 mM). 16 g L^− 1^ agar was used for solid medium. Antibiotics were added to the cultures at the following concentrations (µg mL^− 1^): streptomycin, 500; tetracycline, 2.

**Table 1:** Bacterial strains and plasmids used in this work. Tc, tetracycline

*R. leguminosarum* bv. *viciae* 3841 strain (plasmid)	Description of the strain	Description of the plasmid	Resistance	Source
LMB542 (pIJ11268)	Strain with no *lux* expression used as a negative control.	Plasmid derived from pJP2, containing the *lux* operon with no promoter	Tc	Frederix et al. 2014
D5250 (pIJ11282)	Strain with constitutive *lux* expression used as a positive control.	pIJ11268 with the promoter region of *nptII* cloned upstream of the *luxCDABE* operon	Tc	Frederix et al. 2014
OPS0650 (pOPS0238)	Proline biosensor strain	pIJ11268 with the promoter region of the gene pRL120553 and the divergent gene pRL120552 (*putR*) cloned upstream of the *luxCDABE* operon	Tc	This work

For the construction of the proline biosensor, the promoter region of pRL120553 (605 bp, including the complete upstream regulator pRL120552, *putR*; see gene map in Fig. S1a) was amplified using the primers listed in supporting Table 1 with Phusion High-Fidelity DNA Polymerase (Thermo Fisher). Fragments were purified and double digested with *Kpn*I (at the 5’ end) and *Bam*HI (at the 3’ end). Restriction fragments were cloned in front of the *luxCDABE* operon in the lux biosensor vector pIJ11268 (Frederix et al. 2014) to generate the proline biosensor plasmid pOPS0238 (Fig. S1b). As a positive control, the neomycin promoter producing constitutive luminescence was cloned upstream of *lux* genes into the same plasmid, generating pIJ11282. The promoterless lux vector pIJ11268 was used as a negative control. Plasmids were transferred into wild-type *R. leguminosarum* bv. *viciae* 3841 by triparental mating as previously described (Pini et al. 2017). All plasmids are available from Addgene (https://www.addgene.org).

### Free-living assays

Bacterial strains were grown for 3 days on an UMA (30 mM sodium pyruvate and 10 mM ammonium chloride) slope, resuspended in UMS without carbon or nitrogen and washed three times. Then cells were grown in UMS with 30 mM sodium pyruvate and 10 mM ammonium chloride with antibiotics for 16 h. These cultures were then used as inocula for subsequent induction experiments during 3 h. In these experiments, cells were grown in 10 mL UMS with different supplements (as specified in the corresponding figure). Luminescence (expressed as relative luminescence units, RLU) and OD_600_ were measured using the GloMax-Multi + Detection System (Promega). Specific luminescence was calculated as RLU per OD_600_.

### Plant growth conditions

Pea (*Pisum sativum* var. Avola) seeds were surface sterilized and germinated on distilled water agar (0.8%, w/v) plates for 5 days in the dark at room temperature. Seedlings were then transferred to 13-cm square Petri plates containing Fahräeus agar (Somasegaran et al. 1994) covered with sterile filter paper (one seedling per plate), as previously described (Pini et al. 2017). Seedlings were then inoculated with the corresponding bacterial strains by pouring liquid inoculum to the roots, adjusted to an OD_600_ equivalent to 2 × 10^7^ CFU/per root. Plates were closed with the lid and covered with aluminium foil to prevent exposure of roots to light. Plants were grown in a growth chamber under controlled environmental conditions (23 °C temperature, 16-h/8-h day/night cycle).

### Application and physiological characterisation of water deficit and salt stress

Seven days after inoculation, plants were transferred to fresh Fahräeus agar plates. For control plants, plates were replaced every three days to keep adequate moisture and nutrient levels. To generate water-deficit conditions, plants were maintained in the same plates for seven days so that water was progressively depleted. For the salt stress treatment, plants were transferred to Fahräeus plates containing 150 mM NaCl at 15 dpi for 24 h. In both cases, plants were transferred to fresh Fahräeus plates at 16 dpi and further grown for 5 days (recovery). Plates were analysed at 4, 7, 10, 13, 15, and then daily until 21 dpi.

To establish the effect of water-deficit and salt stress on plants, stomatal conductance, leaf water potential (Ψ_leaf_) and net photosynthesis were measured 2 h after the beginning of photoperiod in the youngest fully expanded leaf. Stomatal conductance and net photosynthesis were measured with a portable open system mode (model LCpro+; ADC BioScientific Ltd.) using an ADC PLC-7504 leaf chamber. Ψ_leaf_ was measured using a pressure chamber (Scholander et al. 1965). Symbiotic nitrogen fixation was measured as apparent nitrogenase activity (ANA) according to the method described by Witty and Minchin (1998). H_2_ evolution of intact plants was measured in an open flow-through system under N_2_/O_2_ (79%/21%) using an electrochemical H_2_ sensor (Qubit Systems). The H_2_ sensor was calibrated with high purity gases using a gas mixer flowing at the same rate as the sampling system (500 mL min^− 1^).

### Image acquisition

Plates were photographed using a NightOWL camera (Berthold Technologies) as previously described (Pini et al. 2017). Briefly, CCD images (1,024 by 1,024 pixels) of light output were exposed for 120 s and analysed with the imaging software IndiGO (Berthold Technologies). Data are expressed as counts per second (cps) or as the ratio of luminescence to surface (cps mm^− 2^).

## Results

### The proline biosensor OPS0650 is able to detect concentrations of proline in the nanomolar range

The gene pRL120553 (hypothetical protein) and pRL120554 (*putA*) was shown to be induced 16-fold and 2-fold respectively in *R. leguminosarum* bv. *viciae* 3841 when grown in the presence of proline (Ramachandran et al. 2011). The gene *putA* encodes the putative multifunctional proline utilisation enzyme A (PutA), which combines ProDH and P5CDH activities, responsible for proline catabolisation in gram-negative bacteria (Liu et al. 2017). pRL120553 codes for a protein of unknown function and protein-BLAST of the predicted protein analysis do not show significant homology with previously described proteins. The gene *putR*, a putative AsnC family transcriptional regulator (pRL120552) is located upstream of pRL120553. These genes are located very close to one another, with intergenic regions around 50 nt each (Fig. S1a). Thus, to generate a proline biosensor strain, a 605-bp region upstream of pRL120553 from *R. leguminosarum* bv. *viciae* 3841 was cloned into the lux vector pIJ11268 in front of the *lux* operon (Fig. S1b). The fragment cloned included the putative transcriptional regulator *putR*, since, from our experience, including the divergent regulator has been shown to improve the sensitivity of the promoter fusions (Pini et al. 2017). Transformation of *R. leguminosarum* bv. *viciae* 3841 with this plasmid generated the proline biosensor strain OPS0650 (Table 1).

**Fig. 1:**
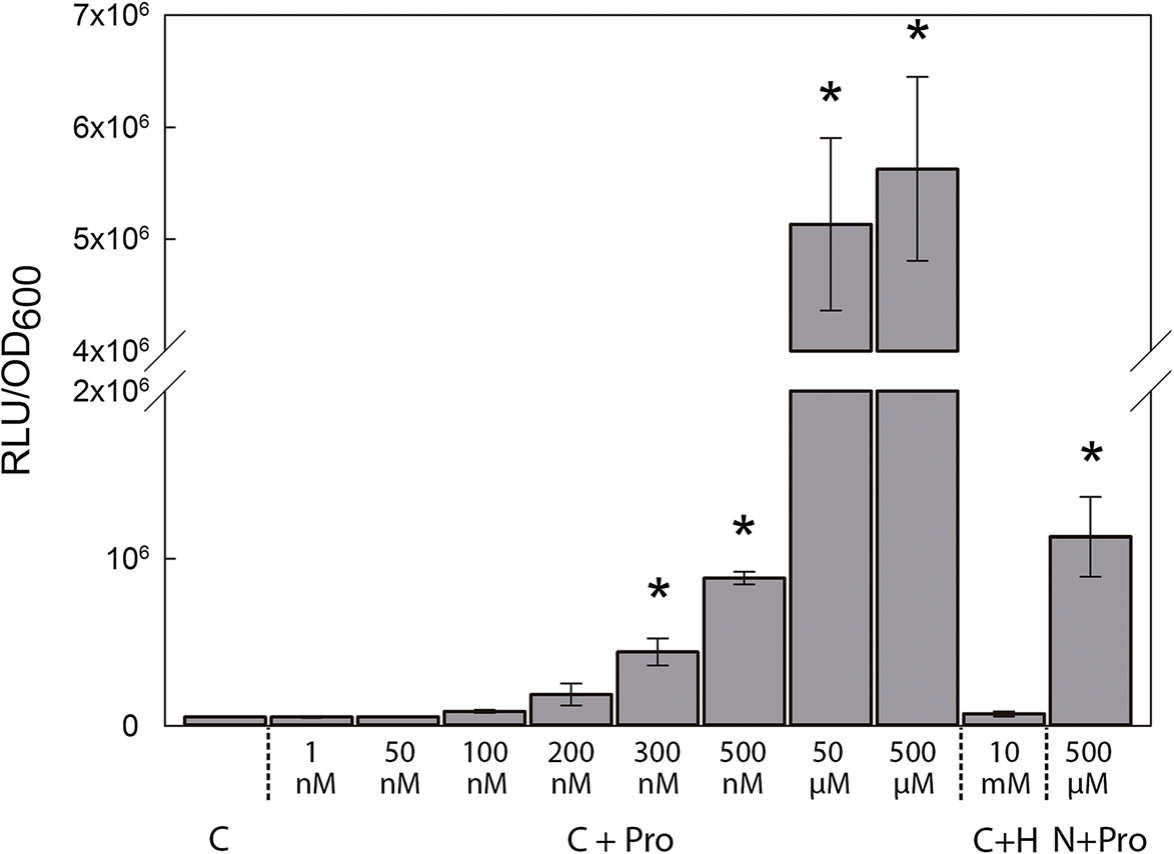
Specificity and sensitivity of the proline biosensor strain OPS0650. Luminescence values are expressed as relative luminescence units (RLU) per optical density at 600 nm (OD_600_). Cells were grown using different combinations of universal minimal salts (UMS) media. C stands for control growth media containing UMS + 30 mM pyruvate + 10 mM ammonium chloride (NH_4_Cl); C + Pro, control medium supplemented with various concentrations of proline as stated; C + H, control medium + 10 mM L-4-hydroxyproline; N + Pro, UMS medium + 10 mM NH_4_Cl + 500 µM Pro. Values represent mean ± SE from two independent experiments in the case of nM proline concentrations and three independent experiments in the other measurements. An asterisk (*) indicates significant differences from C (Student´s *t*-test at p ≤ .05)

To determine the specificity of the induction of *lux* expression in the biosensor, bacterial cells were grown in UMS supplemented with different compounds and luminescence was measured after 3 h of incubation (Fig. 1). Specific luminescence data are represented as relative luminescence units per optical density at 600 nm (RLU/OD_600_) to account for the differences in bacterial growth. In all tests, strain LMB542 containing the empty vector pIJ11268 and the strain D5250 with constitutive *lux* expression were included as negative and positive controls, respectively. The proline biosensor showed luminescence values 86-fold higher in the presence of 500 µM of proline than those to cells grown in control medium. Cells grown in media supplemented with 10 mM of L-4-hydroxyproline, a closely related non-proteinogenic amino acid, presented luminescence values comparable to those of a culture grown in the absence of proline, suggesting that the luminescence recorded is specific for proline. When pyruvate was eliminated from the media, the luminescence showed a decline, yet was still 17-fold higher than that of cells grown in the absence of the amino acid (Fig. 1).

To define the limit of detection of the biosensor, the strain was incubated in UMS media supplemented with different proline concentrations ranging from 1 nM to 500 µM (Fig. 1). The biosensor was able to detect concentrations of proline as low as 300 nM, showing a significant specific luminescence of around 4.42 × 10^5^ ± 0.82 × 10^5^ RLU/OD_600_. Increases in the concentration of proline over 50 µM did not produce significant increments in luminescence emission, maintaining luminescence values of approximately 5.63 × 10^6^ ± 8.22 × 10^5^ RLU/OD_600_.

### *In vivo* monitorisation of proline in root exudates and nodules

**Fig. 2:**
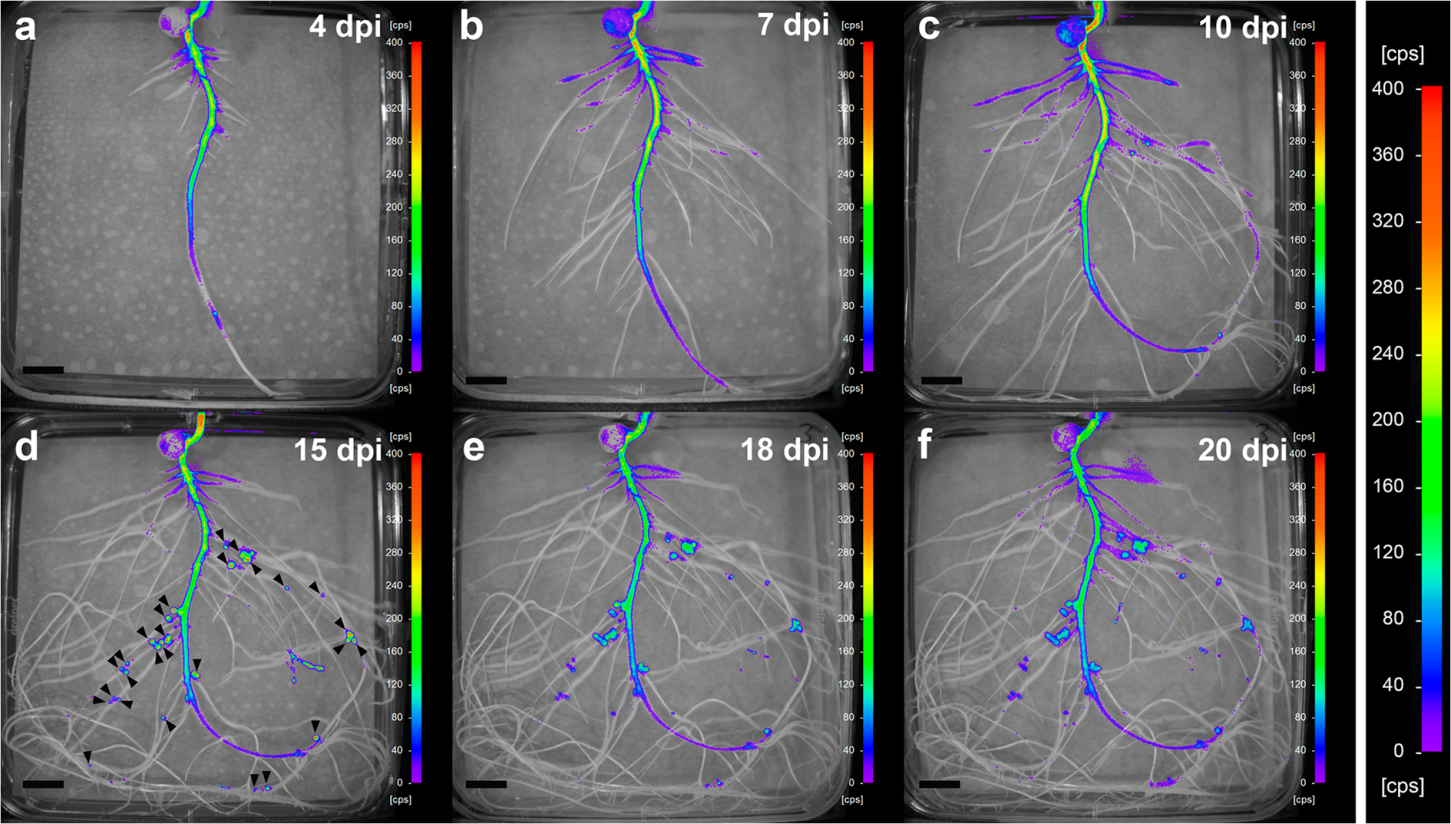
*In vivo* temporal and spatial expression of the proline biosensor strain in pea roots and nodules. Images are representative of plates corresponding to 5 biological replicates and were acquired at 4 **(a)**, 7 **(b)**, 10 **(c)**, 15 **(d)**, 18 **(e)**, and 20 **(f)** dpi. Nodules were visible to the naked eye at around 10 dpi. Arrowheads were added in image (d) to show nodule position. Note that the same scale has been used in all images to facilitate comparisons. Scale bar, 1 cm

We then analysed the spatial and temporal expression of the *lux* reporter in plants inoculated with the biosensor strain during root growth and nodule development. Pea plants were inoculated with either the biosensor strain OPS0650 or the negative and positive control strains LMB542 and D5250, respectively. Luminescence was measured at different time points from 4 to 20 dpi (Fig. 2). We noted that the hypocotyl showed background luminescence most likely due to the presence of chlorophyll in the upper part of the root in proximity to the light. Thus, to measure luminescence in roots we selected the area of the root corresponding to a length of 2–3 cm per root width, located aprox. 2 cm from the hypocotyl to prevent the above-mentioned background luminescence. To calculate luminescence in nodules we used the area corresponding to single nodules. No proline was added to the agar plates and the light output measured in plant roots inoculated with the negative control strain showed average values of 2.90 ± 0.18 cps mm^− 2^ across the experiment (Fig. S2; Fig. S3). Therefore, the differences in luminescence detected can be attributed to either the proline present in root exudates or the proline detected by the differentiated nitrogen-fixing form of rhizobium, the bacteroids, within the infected nodule cells. Four days after inoculation, luminescence was induced mainly on primary roots, particularly in the upper half section of the main root (Fig. 2a). As lateral roots developed, luminescence could also be observed in the oldest lateral roots, although at relatively lower levels (Fig. 2b-f). These discrepancies may be related to the inoculation procedure, which was carried out when plants presented only a main root. At 10 dpi luminescence was detected in nodules (Fig. 2c), reaching maximum intensity values at 15 dpi (Fig. 2d).

### Induction of the proline biosensor occurs after a water-deficit and osmotic stress period

We then employed the luminescence-based proline biosensor strain to analyse the effect of gradual water loss and short-term salt stress on nodulated plants. Water-deficit conditions were created by growing the plants on the same plates for seven days. Doing so, the water content of the agar medium, and therefore, the water available for the plant, was progressively depleted. To monitor the level of the water-deficit stress imposed, we checked the following physiological parameters: leaf water potential (Ψ_leaf_), stomatal conductance, net photosynthesis and symbiotic nitrogen fixation (Table 2). At this stage (16 dpi), Ψ_leaf_ showed a significant decline, with values around − 0.49 ± 0.1 MPa, while control plants maintained at optimal water conditions showed Ψ_leaf_ values of -0.24 ± 0.05 MPa. Similarly, water deficit caused a 66% decline in stomatal conductance and a 36% reduction in photosynthetic rates compared to control plants. Regarding nitrogen fixation, water deficit and salt stress caused a 52 and 67% reduction in the rates of nitrogen fixation, respectively, compared to plants under optimal conditions (Table 2).

**Table 2:** Effect of water deficit and osmotic stress on leaf water potential, stomatal conductance, photosynthesis and ANA of pea plants. Values represent the mean ± SE (7 ≤ n ≤ 10 biological replicates, except in ANA with n = 4). An asterisk (*) indicates significant differences compared to control plants. (Student´s *t*-test at p ≤ .05). NDW, nodule dry weight

Parameter (Units)	Control	Water deficit	Salinity
Stomatal conductance (mol m^− 2^ s^− 1^)	0.09 ± 0.01	0.03 ± 0.01*	0.04 ± 0.01*
Leaf water potential (MPa)	− 0.24 ± 0.05	− 0.49 ± 0.1*	− 0.39 ± 0.03*
Photosynthesis (µmol CO_2_ m^− 2^ s^− 1^)	7.07 ± 0.38	4.52 ± 0.47*	4.73 ± 0.26*
ANA (µmol H_2_ g NDW^− 1^ min^− 1^)	0.54 ± 0.06	0.26 ± 0.01*	0.23 ± 0.07*

In regard to the effects of the different stress treatments on plants inoculated with the proline biosensor, initial experiments showed that the main changes in luminescence were observed in nodules, not in the root exudates. Therefore, analyses of the effect of the stresses are mostly focused on the luminescence of nodules alone. Nevertheless, we also analyzed the variations in luminescence in roots, selecting a region of the main root to facilitate comparisons across treatments. Plants inoculated with the proline biosensor OPS0650 showed significant changes in luminescence when compared with the negative control strain LMB542 (Fig. S3). However, as earlier observed, with the exception of samples at day 16 (24 h after the stress treatments), roots inoculated with biosensor strain did not show significant changes across the experiment.

**Fig. 3:**
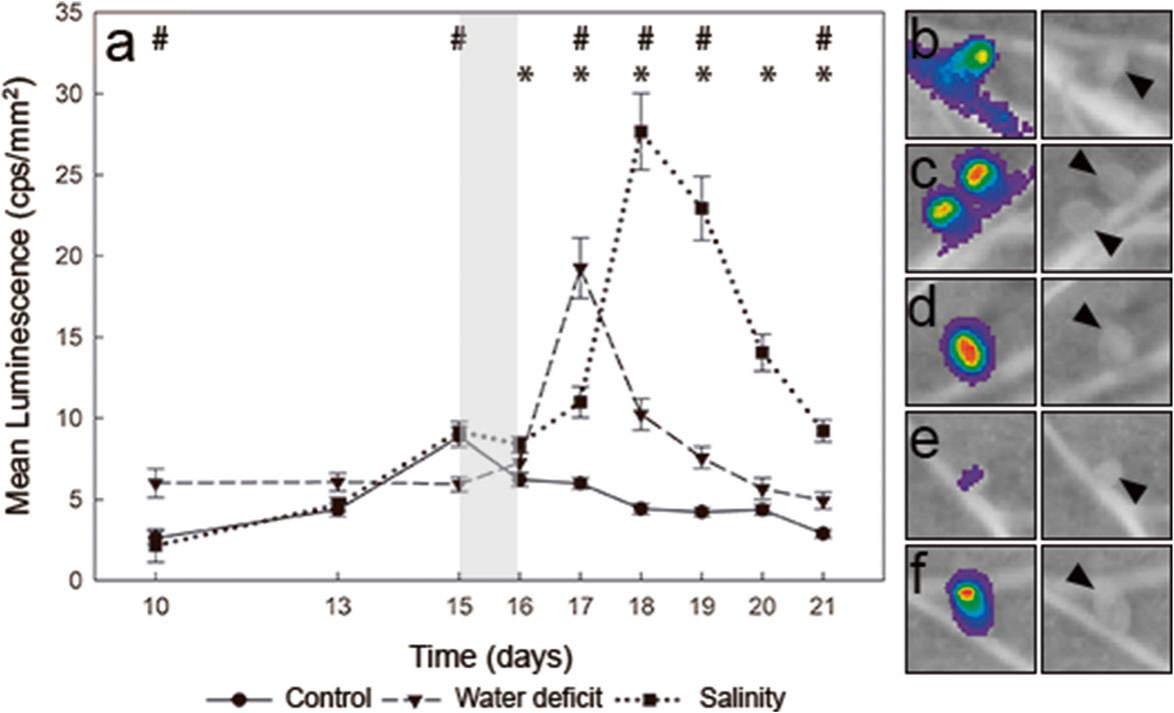
**a***In vivo* monitoring of proline in pea nodules of plants inoculated with the biosensor *R. leguminosarum* bv. *viciae* strain OPS650. Values represent mean luminescence [counts per second (cps) mm^− 2^] from nodules of pea plants inoculated with the proline biosensor. Stress was induced at 15 dpi for 24 h (grey area). For recovery, plants were transferred to fresh plates (optimal growth conditions). Values represent the mean ± SE calculated from 5 biological replicates using the luminescence values of all the nodules present in a plant. An asterisk or a hash sign (* or #) indicate significant differences between salt-stressed or water-deficit plants and control plants, respectively (ANOVA at p < .05, Dunnett T3 post hoc test). (b) to (f) representative images from nodules of pea plants under different conditions, and their corresponding luminescence image. **b** nodules of plants inoculated with the proline biosensor strain OPS0650 under control conditions (17 dpi, scale 0-400 cps); **c** nodules from water-deficit plants at the maximum level of luminescence of the proline biosensor during recovery (17 dpi, scale 0-1100 cps); **d** nodules from salt-stressed plants at the maximum luminescence of the proline biosensor during recovery (18 dpi, scale 0-3000 cps); **e** nodules from plants inoculated with the negative control strain LMB542 (no *lux* expression, 17 dpi, scale 0-400 cps); **f** nodules from plants inoculated with the positive control strain D5250 (constitutive *lux* expression,17 dpi, scale 1200–25200 cps). Arrowheads were added in images (b) to (f) to show nodule position

Regarding the effect of the stresses on nodules, during the period of gradual water loss, the luminescence of nodules was maintained at relatively constant values, suggesting that proline levels did not show significant changes in bacteroids (Fig. 3a, Fig. S4). At 16 dpi, plants were transferred to fresh plates (i.e., optimal water availability) as a recovery treatment. Recovery provoked a rapid increase in the luminescence observed in nodules within the first 24 h, showing a gradual reduction in the following days (Fig. 3a and c, Fig. S4), almost reaching the levels of *lux* expression in nodules of control plants (Fig. 3a and b).

Salt stress was generated by incubating plants on plates containing 150 mM NaCl for one day. Similarly, to the water-deficit experiment, recovery was carried out by transferring the plants to fresh plates under optimal growth conditions, corresponding to the time point 16 dpi. Salinity also had a negative impact on the physiology of the plants, which showed Ψ_leaf_ values of -0.39 ± 0.03 MPa, a 55% decline in stomatal conductance and a 35% reduction in net photosynthesis (Table 2). 24 h after the application of the salt stress there were no significant changes in the level of luminescence of the proline biosensor compared to the luminescence recorded before the onset of the treatment (Fig. 3a, Fig. S5). At 17 dpi, however, luminescence started to increase, reaching its maximum at 18 dpi (Fig. 3a and d, Fig. S5). Subsequently, the levels of luminescence progressively declined, yet were significantly higher than those of control nodules at the end of the experiment (Fig. 3a).

Nodules of plants inoculated with the negative control strain LMB542 showed negligible luminescence levels (Fig. 3e; Fig. S2), while nodules of plants inoculated with D5250, the strain carrying the constitutive *lux* expression plasmid, showed the maximum levels of luminescence (Fig. 3f; Fig. S6).

## Discussion

This work describes a novel lux biosensor for the detection of proline. The biosensor takes advantage of the proline-specific promoter of the gene pRL120553, a gene in close proximity to the gene pRL120554 (*putA*), of *Rhizobium leguminosarum* bv. *viciae* 3841, which were highly expressed during growth in proline. The induction assay showed that this biosensor is very sensitive as it can sense proline at concentrations as low as 300 nM. Also, testing with L-4-hydroxyproline, a close relative of proline, showed that this biosensor is very specific to proline. Currently, this biosensor is housed in *Rhizobium leguminosarum* bv. *viciae* 3841, although it should work in other related rhizobia. This novel construct allows the semi-quantitative estimation of the levels of proline both secreted by roots in the rhizosphere as well as the proline accessible for bacteroids inside nodules. The measurement is carried out in a non-invasive manner, maintaining tissue integrity, and thus, avoiding possible artefacts or degradation issues faced when collecting root exudates or isolating symbiosomes. Additionally, it allows the monitoring of proline levels across time using a simple plate growth assay. Although *lux*-based systems come with their own limitations (i.e., dependence on oxygen, ATP or reducing power; Brodl et al. 2018), this type of *lux*-based biosensor has been successfully used for the *in vivo* monitoring of a number of metabolites including sugars, polyols and organic acids (Pini et al. 2017), as well as signalling compounds such as rhizopines (Geddes et al. 2019).

In terms of root secretion, proline was detected mainly on the primary root and, at later stages, with lateral roots and nodules (Fig. 2, Fig. S2, Fig. S3). This luminescence was shown to be specific of the presence of proline since plants inoculated with the negative control strain showed negligible light output (Fig. S2, Fig. S3). Interestingly, these regions do not correspond to root zones that are preferentially colonised by rhizobia (i.e., the elongation zone), neither co-localise with the secretion of other metabolites such as the amino acid phenylalanine or carbohydrates like sucrose (Pini et al. 2017). This suggests that the composition of the root exudates varies depending on the root zones, an observation that could not be made using classical root exudate extraction approaches.

Symbiotic root nodules represent a strong sink tissue for the plant, requiring the transport of high levels of sucrose to fuel nitrogenase activity and the biosynthesis of a large number of proteins and other biomolecules to maintain the high rates of metabolic activities. Once inside nodules, the major energy source provided by the plant to the bacteroids is in the form of malate (Udvardi et al. 1988; Ronson et al. 1981; Driscoll and Finan 1993). However, the peribacteroid membrane also allows the transport of amino acids, including proline (Udvardi et al. 1990; Zhu et al. 1992). This fact, along with the observation of increased proline degradation activity under drought and salt stress conditions (Kohl et al. 1988), has led to suggest that besides its osmoprotectant role, proline can also be used as a carbon, nitrogen and energy source for bacteroids (Curtis et al. 2004; Kohl et al. 1994). Indeed, exogenous application of proline or inoculation with a strain overexpressing *putA* has been shown to improve nitrogen fixation under drought stress conditions (Kohl et al. 1994; Zhu et al. 1992; van Dillewijn et al. 2001).

The current work shows that proline levels increased within bacteroids during nodule development, reaching a maximum level at 15 dpi. It is also interesting to note that, based on the luminescence detected, proline levels in nodules are significantly higher than those detected in root exudates. However, in contrast to the classical stress-induced accumulation of proline in nodules (Fougere et al. 1991; Gil-Quintana et al. 2013; Larrainzar et al. 2009), proline levels in pea bacteroids did not show significant variations during the stress period, but during the recovery phase. Although there are several factors that could account for these discrepancies such as the duration or the intensity of the stress treatment, it is noteworthy that this is the first time that proline levels are measured in bacteroids within intact nodules (i.e., without disrupting the tissue). Thus, the increase in proline previously reported may account, at least partially, to the proline accumulation in the plant fraction of nodules. In this scenario, recuperation of optimal growth conditions during recovery may lead to a reactivation of bacteroid proline catabolism and/or increased import of proline from the cytosol to the symbiosomes. This activation of proline catabolism upon recovery has, to our knowledge, been so far only described in plants, where the expression of proline dehydrogenase is suppressed during osmotic stress but induced again upon the relief of the stress (Mani et al. 2002; Satoh et al. 2002). Proline uptake has been shown to occur through a diffusive process in bacteroids from alfalfa and soybean nodules (Trinchant et al. 1998; Pedersen et al. 1996; Udvardi et al. 1990). Thus, one possibility is that at least a fraction of the proline accumulated during the stress in the plant fraction catabolised by the bacteroid with a two-fold benefit: to facilitate restoring pre-stress proline levels in the cytosol of infected cells, and to provide an additional source of energy, carbon or nitrogen for bacteroid metabolism. In this regard, in experiments with bacteroids isolated from *Vicia faba* nodules, salt stress produced an accumulation of proline in the peribacteroid space, suggesting that symbiosomes may behave as osmometers to accommodate the osmotic changes occurring in infected cells (Trinchant et al. 1998). Based on results presented here, this could also be the situation in nodules subjected to water deficit. It would be of great interest to combine multiple biosensors driving the expression of, for instance, different fluorescent proteins so that the levels of key metabolites can be simultaneously monitored *in vivo*.

## Electronic supplementary material

Fig. S1 Gene (a) and plasmid map (b) indicating the 605-bp region cloned to construct the lux-based proline biosensor strain OPS0650 in R. leguminosarum bv. viciae 3841 (PNG 4139 kb)

High Resolution Image (TIF 830 kb)

Fig. S2 *In vivo* temporal and spatial expression of negative control strain LMB542 in roots and nodules of pea plants. Luminescence is expressed in counts per second (cps). Images were acquired at 4 (a), 7 (b), 10 (c), 15 (d), 18 (e), and 20 (f) dpi. Arrowheads were added in image (d) to show nodule position. Note that the same scale has been used in all images to facilitate comparisons. Representative images of plates belonging to a time series experiment (n = 5 biological replicates). Scale bar, 1 cm (PNG 6376 kb)

High Resolution Image (TIF 25513 kb)

Fig. S3 *In vivo* monitoring of proline in roots of plants inoculated with the biosensor *R. leguminosarum* bv. *viciae* OPS650 or LMB542 strains. Values represent mean luminescence (cps mm− 2) from roots of pea plants inoculated with the proline biosensor and the negative control. Stress was induced at 15 dpi for 24 h (grey area). Values represent the mean ± SE calculated from 4 biological replicates using the luminescence values of a section of the main root (section of 2–3 cm long per root width, 2 cm away from hypocotyl). An asterisk (*) indicates significant differences between water and salt-stressed plants compared to plants inoculated with the negative control strain; a hash (#) indicates significant differences between control plants and the negative control; a square (▪) indicates significant differences between salt-stressed and water-deficit plants compared to control plants (ANOVA at p < .05, LSD post hoc test) (PNG 1596 kb)

High Resolution Image (TIF 6396 kb)

Fig. S4 *In vivo* temporal and spatial expression of the proline biosensor strain in roots and nodules of water-stressed pea plants. Luminescence is expressed in counts per second (cps). Images were acquired at 15 (a), 16 (b), 17 (c), 18 (d), 19 (e), and 20 (f) dpi. Arrowheads were added in image (a) to show nodule position. Note that the same scale has been used in all images to facilitate comparisons. Representative images of plates belonging to a time series experiment (n = 5 biological replicates). Scale bar, 1 cm (PNG 6376 kb)

High Resolution Image (TIF 25513 kb)

Fig. S5 *In vivo* temporal and spatial expression of the proline biosensor strain in roots and nodules of salt-stressed pea plants. Luminescence is expressed in counts per second (cps). Images were acquired at 15 (a), 16 (b), 17 (c), 18 (d), 19 (e), and 20 (f) dpi. Arrowheads were added in image (a) to show nodule position. Note that the same scale has been used in all images to facilitate comparisons. Representative images of plates belonging to a time series experiment (n = 5 biological replicates). Scale bar, 1 cm (PNG 6376 kb)

High Resolution Image (TIF 25514 kb)

Fig. S6 *In vivo* spatial expression of the positive control strain D5250 constitutively expressing the *lux* construct in roots and nodules of pea plants. Luminescence is expressed as counts per second (cps). Panels (a) and (b) show the same plant analyzed using two different detection thresholds: 200 and 1200 cps, respectively. Images were acquired at 17 dpi. Arrowheads were added in image (a) to show nodule position. Representative images of plates belonging to a time series experiment (n = 5 biological replicates). Scale bar, 1 cm (PNG 1596 kb)

High Resolution Image (TIF 6397 kb)

Table S1 (DOCX 26 kb)

## Abbreviations

ANA: apparent nitrogenase activity
CFU: colony-forming units
cps: counts per second
dpi: days post inoculation
P5C: pyrroline-5-carboxylate
P5CDH: delta-1-pyrroline-5-carboxylate dehydrogenase
ProDH: proline dehydrogenase
RLU: relative luminescence units
OD_600_: optical density at 600 nm
UMA: universal minimal agar
UMS: universal minimal salts
