# Supplementary figures and images for: A novel biosensor to monitor proline in pea root exudates and nodules under osmotic stress and recovery
Source: Plant Soil. 2020 Jun 4;452(1):413–22. doi: 10.1007/s11104-020-04577-2 (PMC7371648; doi:10.1007/s11104-020-04577-2)

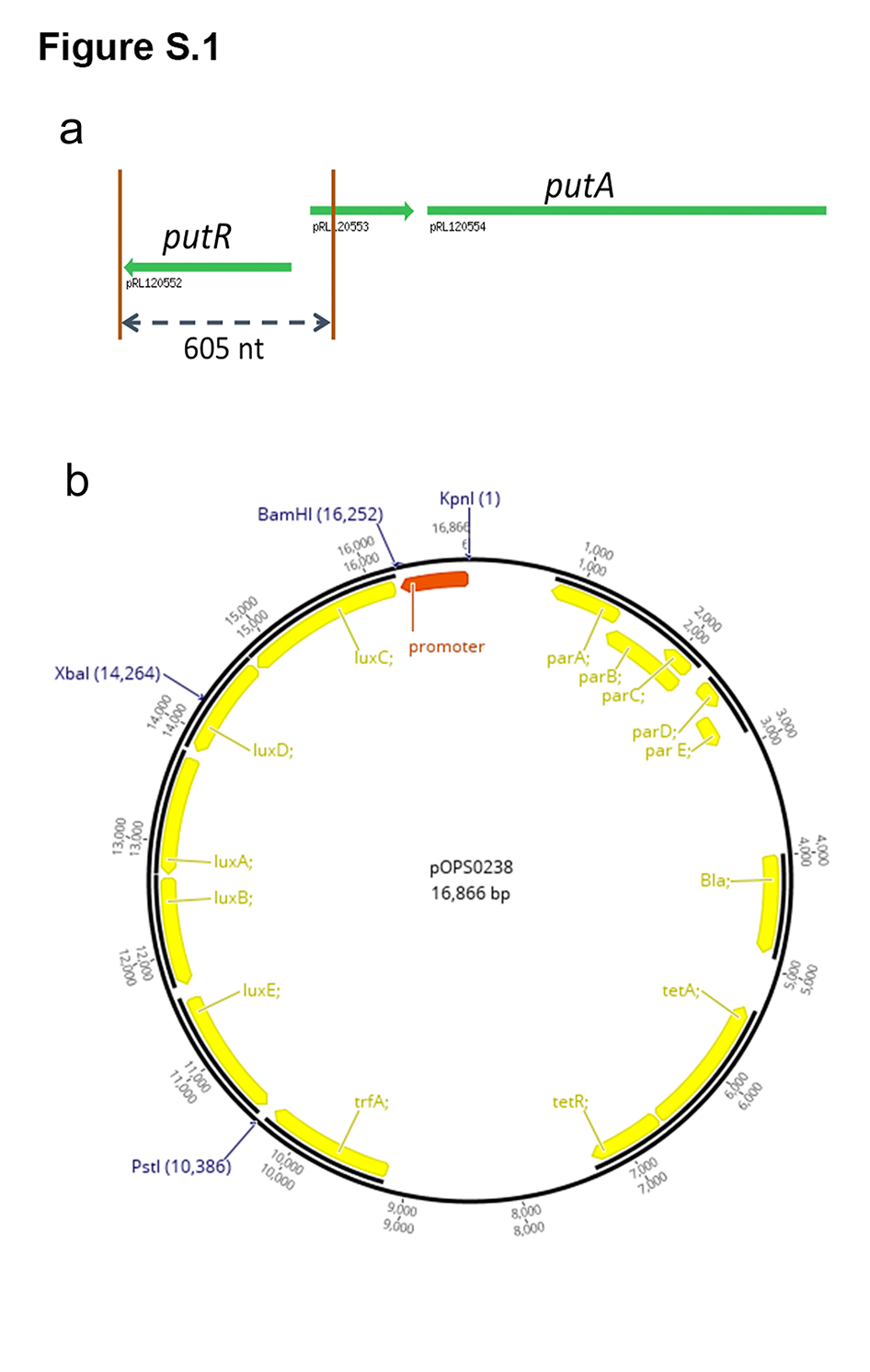

Supplement: Supplementary file 1 — Gene (a) and plasmid map (b) indicating the 605-bp region cloned to construct the lux-based proline biosensor strain OPS0650 in R. leguminosarum bv. viciae 3841 (PNG 4139 kb) [file 11104_2020_4577_Fig4_ESM.png]

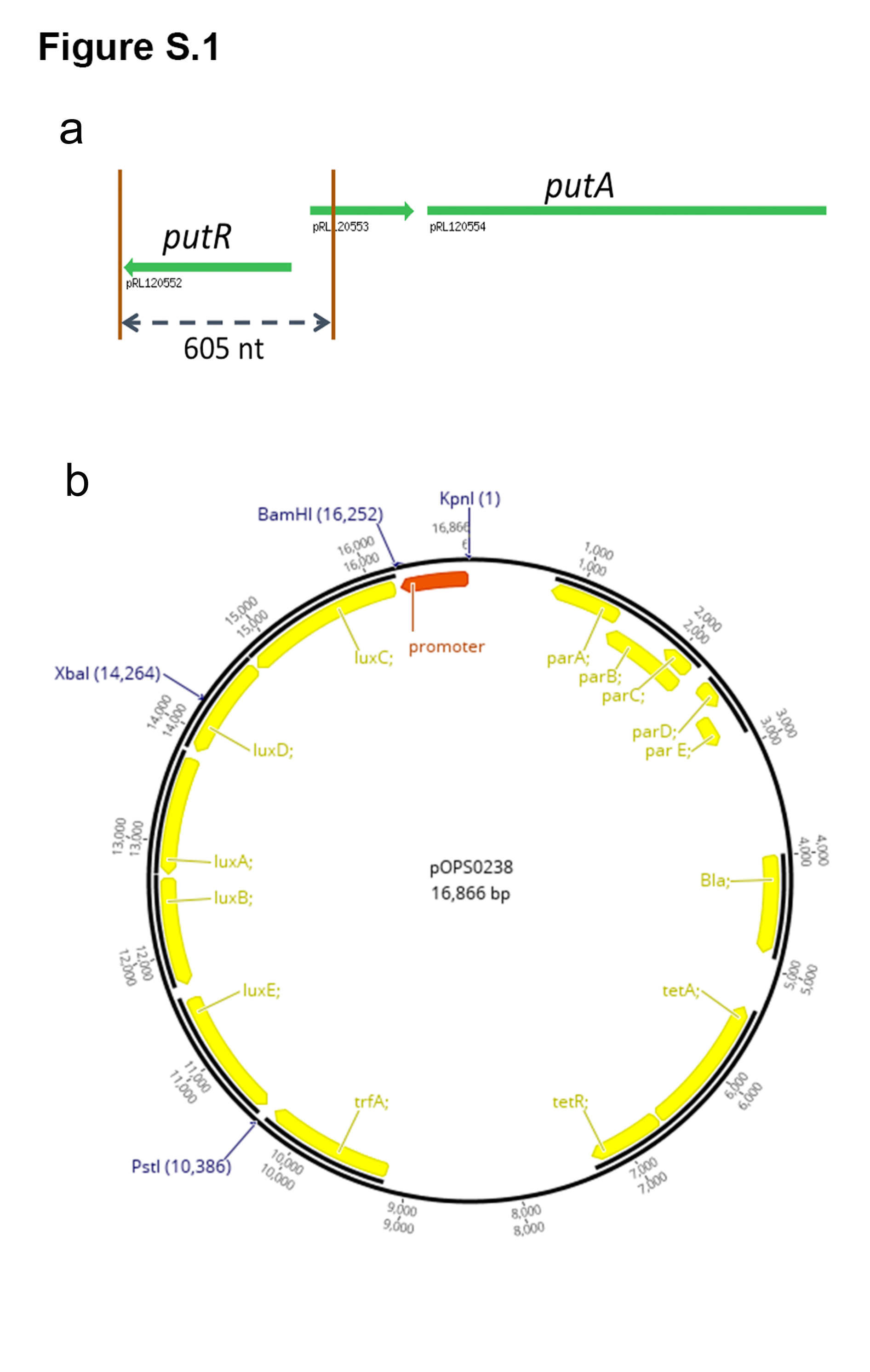

Supplement: Supplementary file 2 — High Resolution Image (TIF 830 kb) [file 11104_2020_4577_MOESM1_ESM.tif]

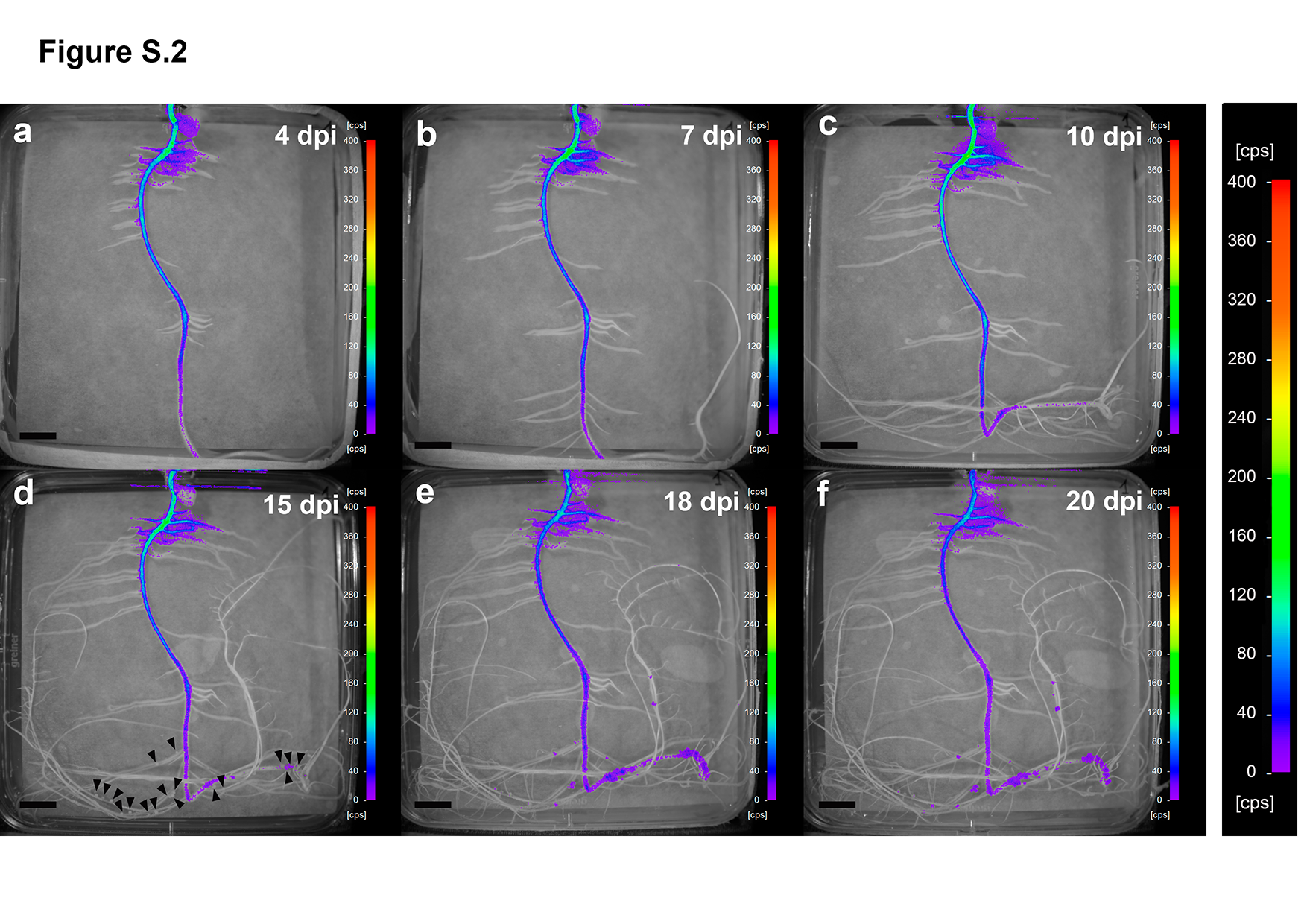

Supplement: Supplementary file 3 — In vivo temporal and spatial expression of negative control strain LMB542 in roots and nodules of pea plants. Luminescence is expressed in counts per second (cps). Images were acquired at 4 (a), 7 (b), 10 (c), 15 (d), 18 (e), and 20 (f) dpi. Arrowheads were added in image (d) to show nodule position. Note that the same scale has been used in all images to facilitate comparisons. Representative images of plates belonging to a time series experiment (n = 5 biological replicates). Scale bar, 1 cm (PNG 6376 kb) [file 11104_2020_4577_Fig5_ESM.png]

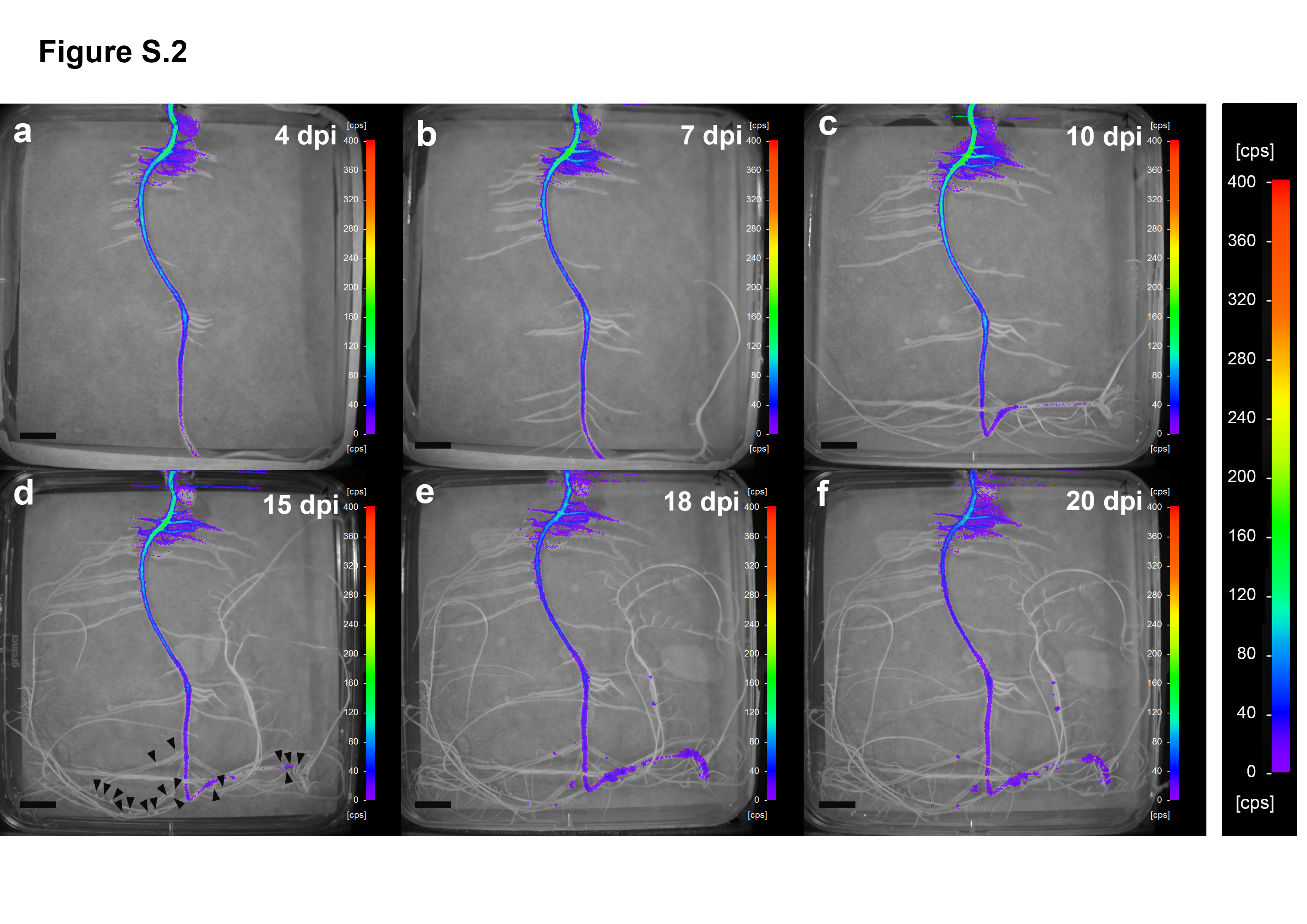

Supplement: Supplementary file 4 — High Resolution Image (TIF 25513 kb) [file 11104_2020_4577_MOESM2_ESM.tif]

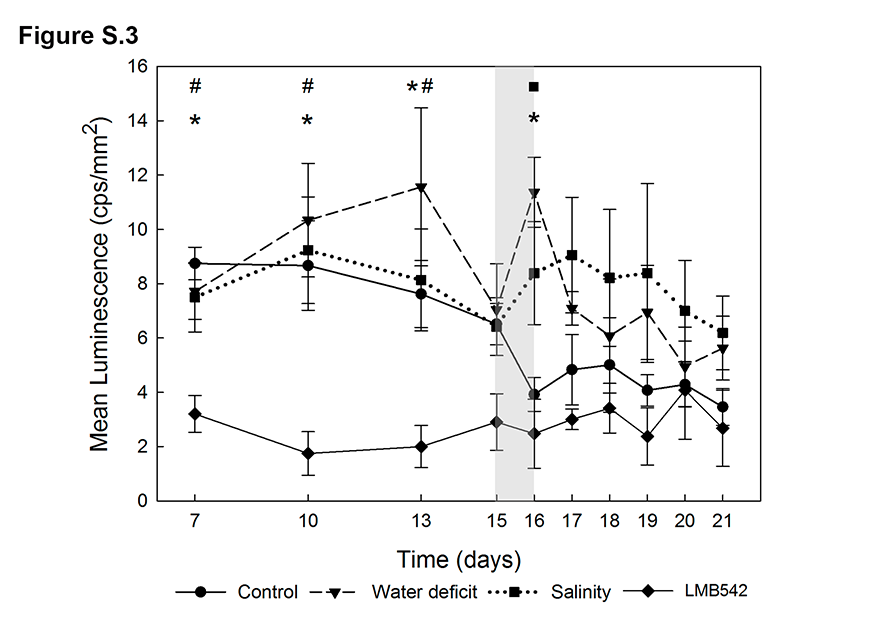

Supplement: Supplementary file 5 — In vivo monitoring of proline in roots of plants inoculated with the biosensor R. leguminosarum bv. viciae OPS650 or LMB542 strains. Values represent mean luminescence (cps mm− 2) from roots of pea plants inoculated with the proline biosensor and the negative control. Stress was induced at 15 dpi for 24 h (grey area). Values represent the mean ± SE calculated from 4 biological replicates using the luminescence values of a section of the main root (section of 2–3 cm long per root width, 2 cm away from hypocotyl). An asterisk (*) indicates significant differences between water and salt-stressed plants compared to plants inoculated with the negative control strain; a hash (#) indicates significant differences between control plants and the negative control; a square (▪) indicates significant differences between salt-stressed and water-deficit plants compared to control plants (ANOVA at p < .05, LSD post hoc test) (PNG 1596 kb) [file 11104_2020_4577_Fig6_ESM.png]

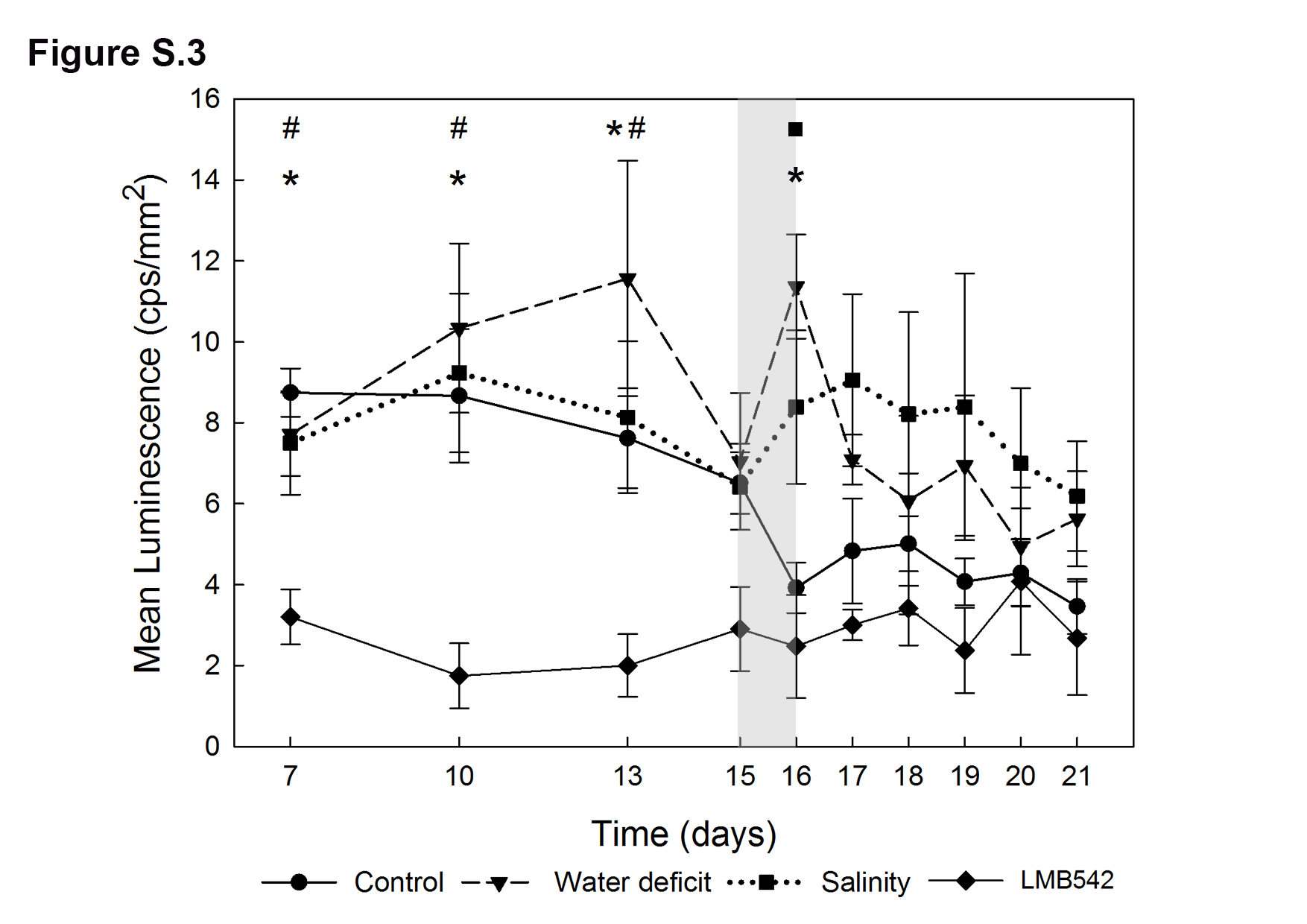

Supplement: Supplementary file 6 — High Resolution Image (TIF 6396 kb) [file 11104_2020_4577_MOESM3_ESM.tif]

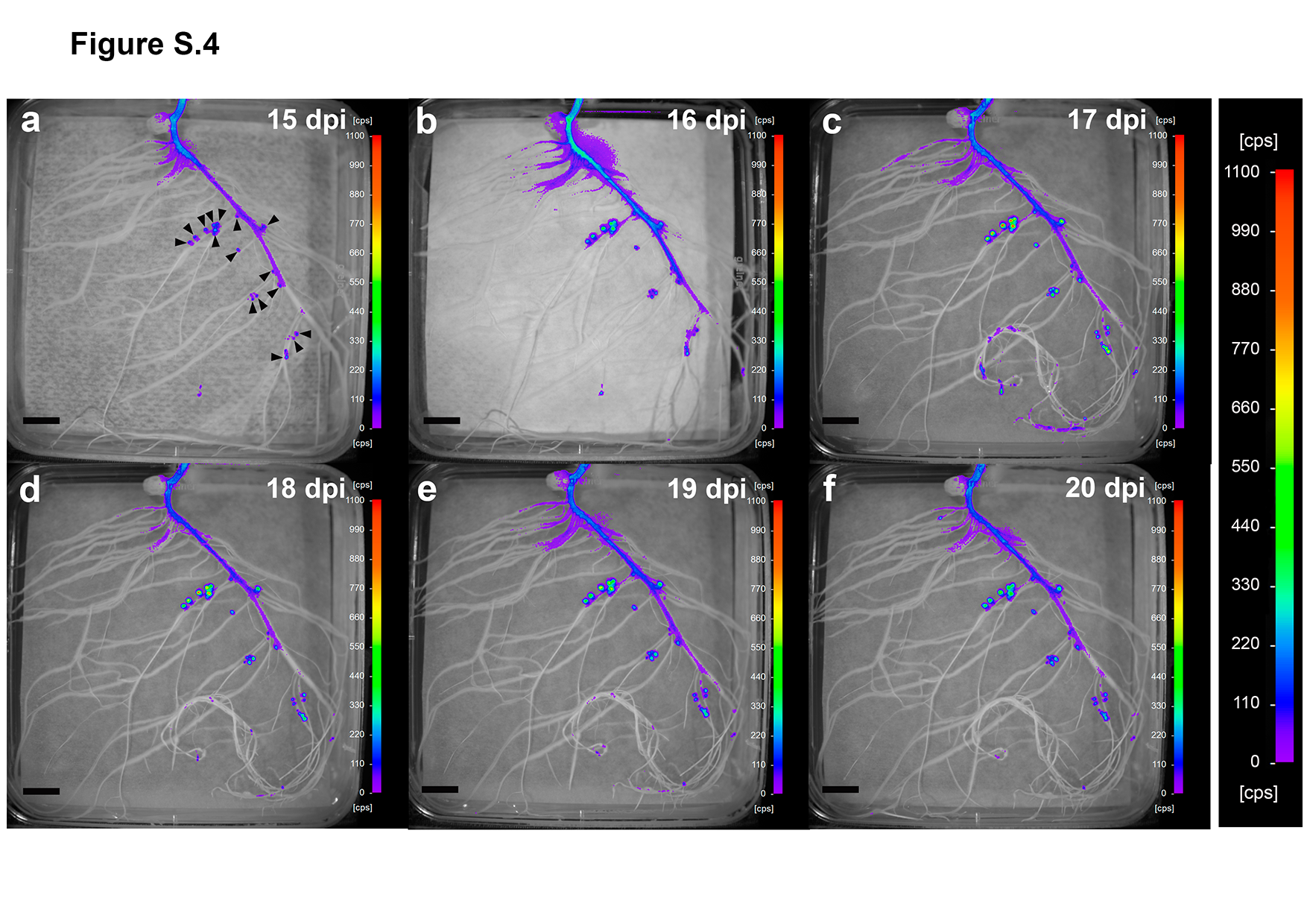

Supplement: Supplementary file 7 — In vivo temporal and spatial expression of the proline biosensor strain in roots and nodules of water-stressed pea plants. Luminescence is expressed in counts per second (cps). Images were acquired at 15 (a), 16 (b), 17 (c), 18 (d), 19 (e), and 20 (f) dpi. Arrowheads were added in image (a) to show nodule position. Note that the same scale has been used in all images to facilitate comparisons. Representative images of plates belonging to a time series experiment (n = 5 biological replicates). Scale bar, 1 cm (PNG 6376 kb) [file 11104_2020_4577_Fig7_ESM.png]

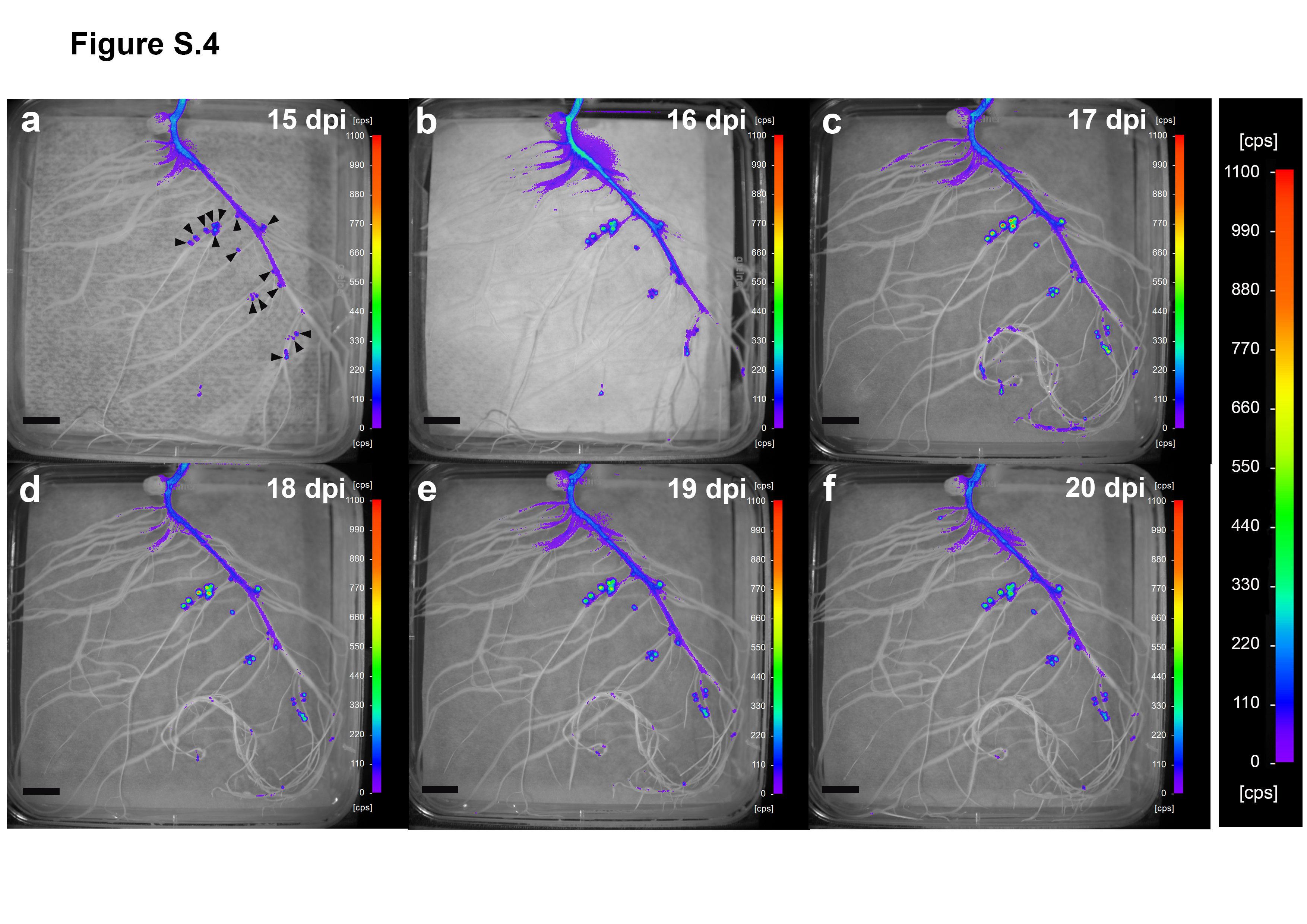

Supplement: Supplementary file 8 — High Resolution Image (TIF 25513 kb) [file 11104_2020_4577_MOESM4_ESM.tif]

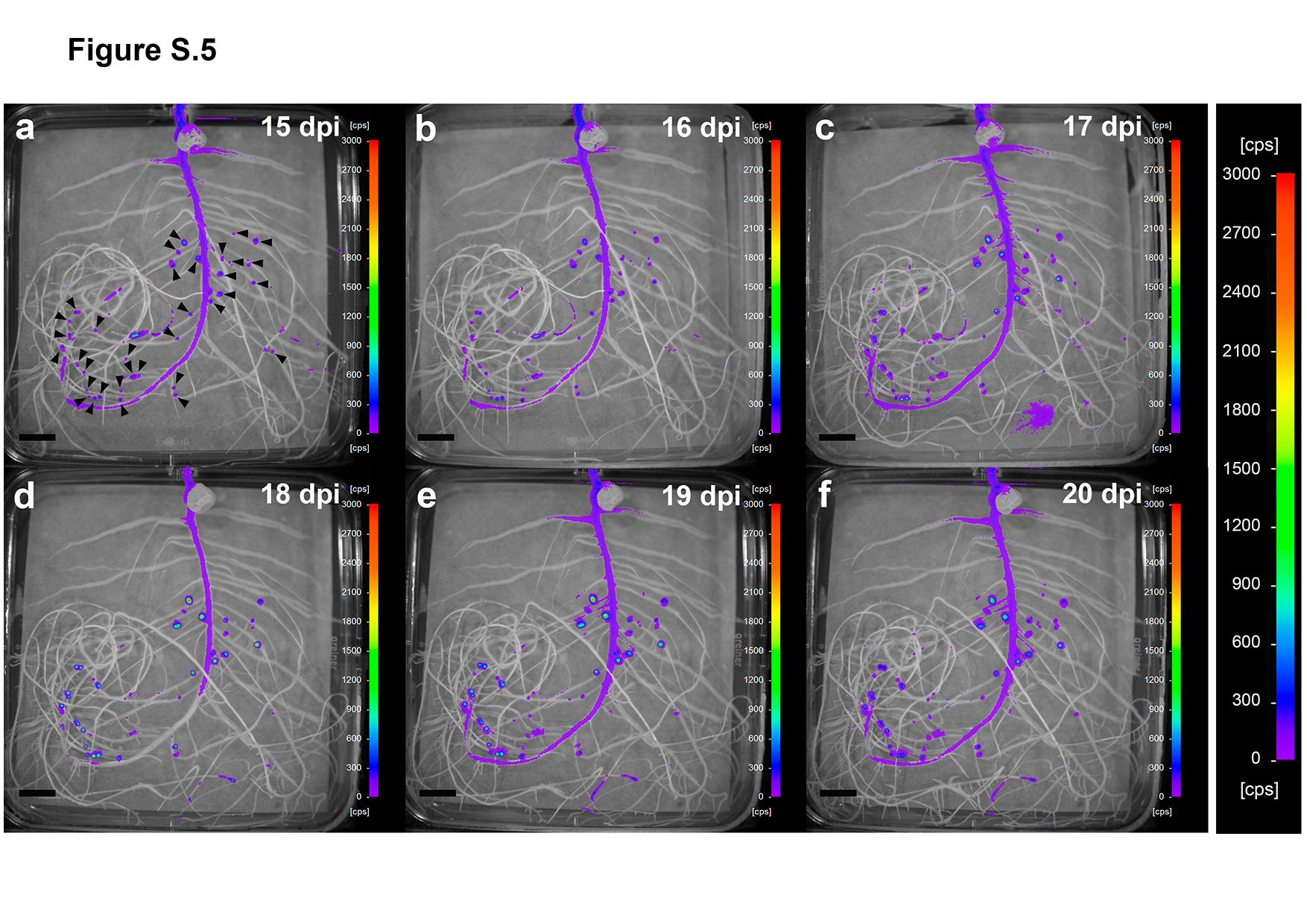

Supplement: Supplementary file 9 — In vivo temporal and spatial expression of the proline biosensor strain in roots and nodules of salt-stressed pea plants. Luminescence is expressed in counts per second (cps). Images were acquired at 15 (a), 16 (b), 17 (c), 18 (d), 19 (e), and 20 (f) dpi. Arrowheads were added in image (a) to show nodule position. Note that the same scale has been used in all images to facilitate comparisons. Representative images of plates belonging to a time series experiment (n = 5 biological replicates). Scale bar, 1 cm (PNG 6376 kb) [file 11104_2020_4577_Fig8_ESM.png]

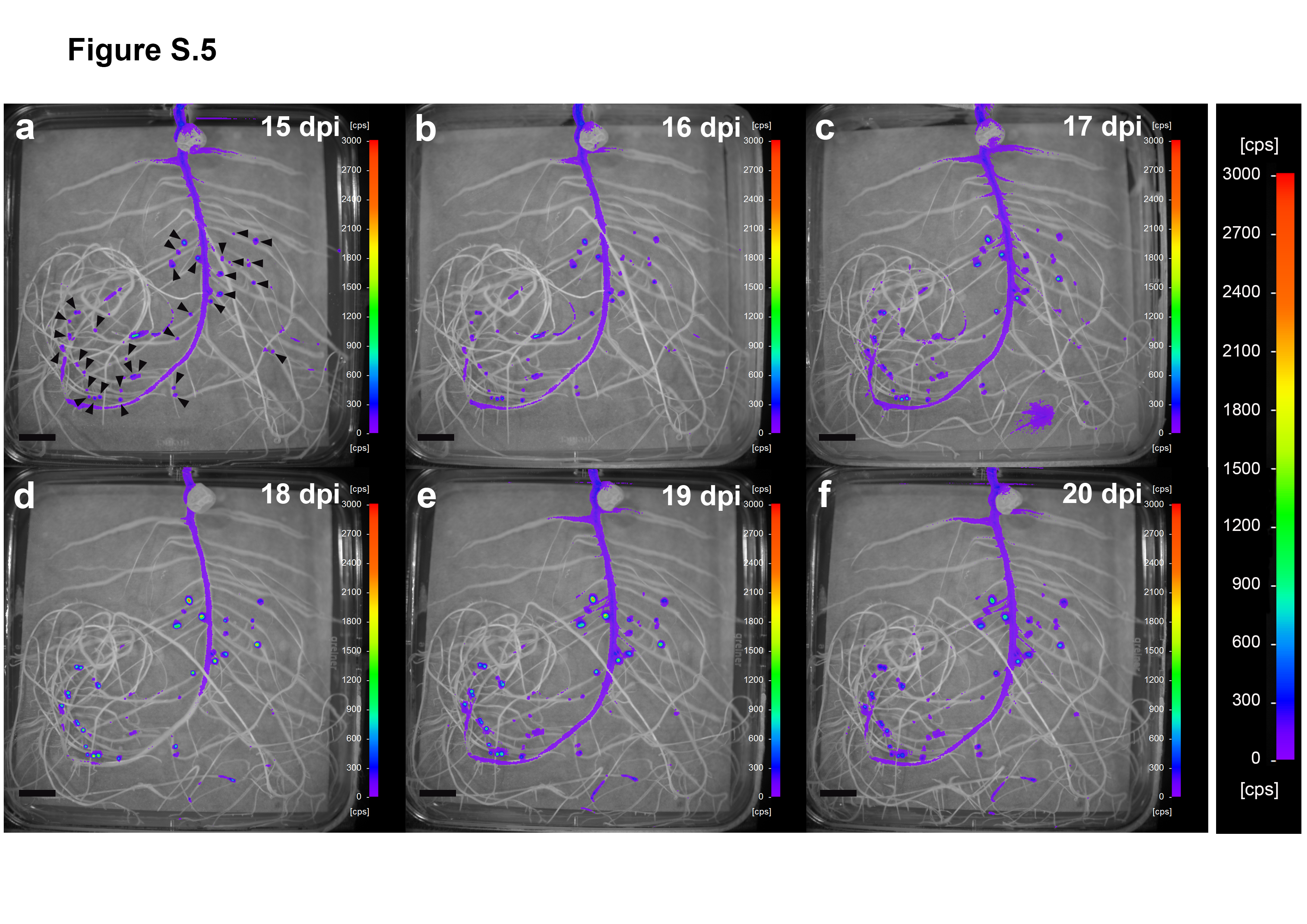

Supplement: Supplementary file 10 — High Resolution Image (TIF 25514 kb) [file 11104_2020_4577_MOESM5_ESM.tif]

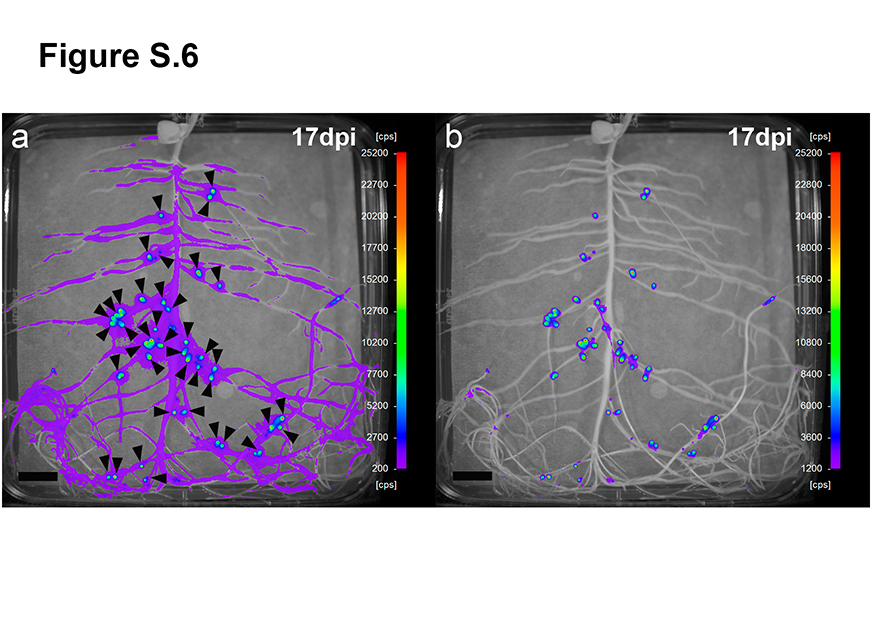

Supplement: Supplementary file 11 — In vivo spatial expression of the positive control strain D5250 constitutively expressing the lux construct in roots and nodules of pea plants. Luminescence is expressed as counts per second (cps). Panels (a) and (b) show the same plant analyzed using two different detection thresholds: 200 and 1200 cps, respectively. Images were acquired at 17 dpi. Arrowheads were added in image (a) to show nodule position. Representative images of plates belonging to a time series experiment (n = 5 biological replicates). Scale bar, 1 cm (PNG 1596 kb) [file 11104_2020_4577_Fig9_ESM.png]

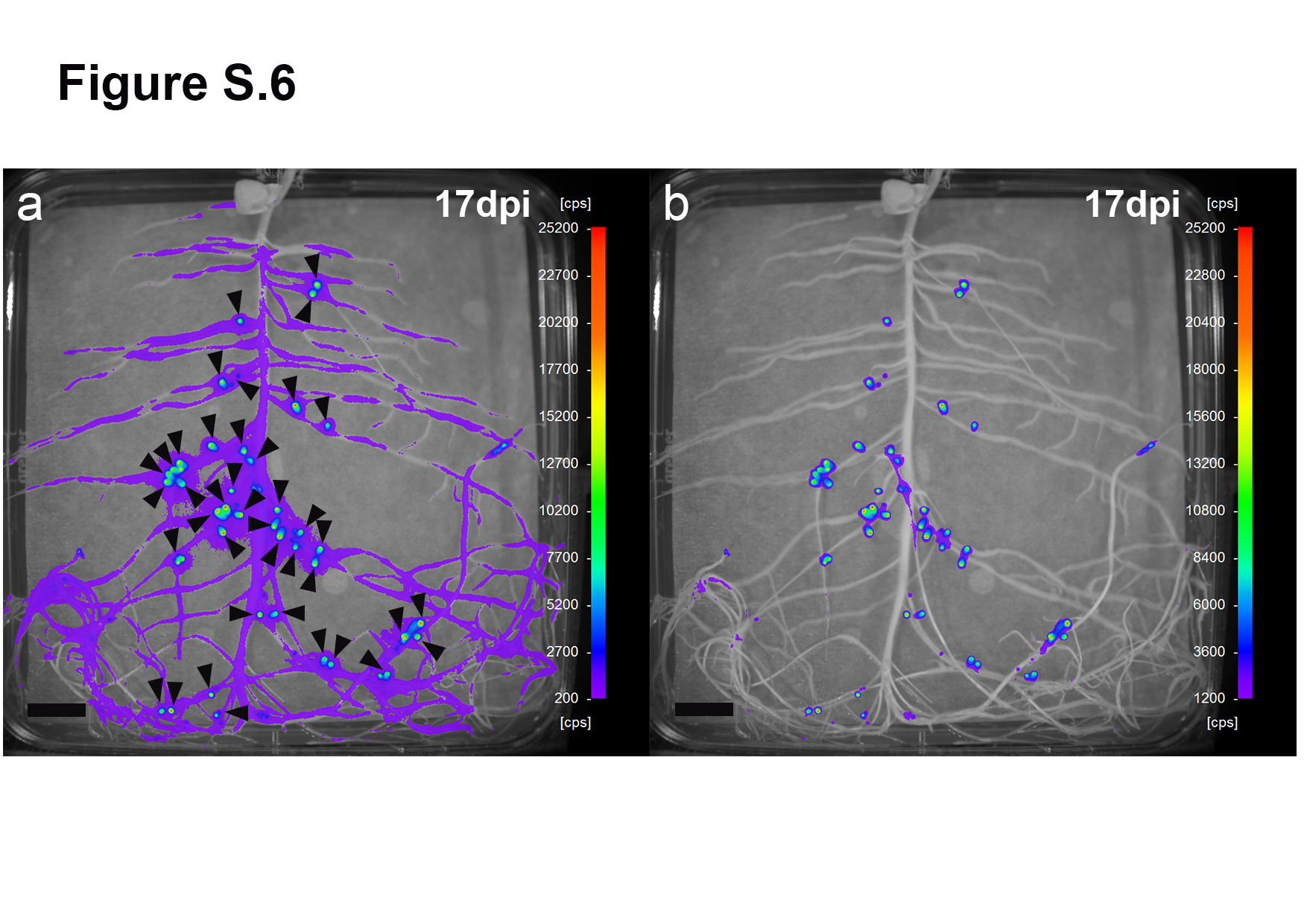

Supplement: Supplementary file 12 — High Resolution Image (TIF 6397 kb) [file 11104_2020_4577_MOESM6_ESM.tif]
