# Supplementary material for: A novel biosensor to monitor proline in pea root exudates and nodules under osmotic stress and recovery
Source: Plant Soil. 2020 Jun 4;452(1):413–22. doi: 10.1007/s11104-020-04577-2 (PMC7371648; doi:10.1007/s11104-020-04577-2)
Supplement: Supplementary file 13 — (DOCX 26 kb) [file 11104_2020_4577_MOESM7_ESM.docx]

**Table S1** Primers used in this work

| **Primer** | **Sequence** | **Description** | **Restriction site** | **Source** | **Biosensor** |
| --- | --- | --- | --- | --- | --- |
| oxp0648 | TTTTGGTACCCGTCATAGCCTCCCACGGAT | Sense primer for region upstream of pRL120553 including divergent gene pRL120552 | *Kpn*I | This work | Proline |
| oxp0649 | TTTTGGATCCCTATCATAATCACAAATCGCAATCTCA | Antisense primer for region upstream of pRL120553 including divergent gene pRL120552 | *Bam*HI | This work | Proline |
